# Frequency of thrombophilia associated genes variants: population-based study

**DOI:** 10.1186/s12881-020-01136-5

**Published:** 2020-10-09

**Authors:** Natalia Wawrusiewicz-Kurylonek, Adam Jacek Krętowski, Renata Posmyk

**Affiliations:** 1 Department of Endocrinology, Diabetology and Internal Medicine, Medical University of Bialystok, ul. M. Curie-Skłodowskiej 24A, 15-276 Białystok, Poland; 2 Podlaskie Center of Clinical Genetics “Genetics”, Bialystok, Poland; 3 Department of Clinical Genetics, Medical University in Bialystok, Bialystok, Poland

## Abstract

**Background:**

Thrombophilia is a hypercoagulable state that may have a genetic basis (inherited) or can be acquired. It is a multifactorial condition and only the mutual interactions between the environment and genes may lead to the development of clinical manifestation. This state is the main factor promoting venous (rarely arterial) thromboembolism (VTE). Inherited thrombophilia is mainly associated with two pathogenic variants in the V coagulation factor (*FV*) and the prothrombin (*FII*) genes. The aim of our study was to evaluate the frequency of two pathogenic variants in *FII* and *FV* genes as inherited thrombophilia factors in a group within the Polish population in comparison with other described populations.

**Methods:**

All studied groups consisted of 633 unrelated patients aged between 18 and 70. Individuals in the research group come from the Podlasie region of Poland. Genotyping of FII and FV variants was performed using the 7900HT Fast Real-Time PCR System and were genotyped by TaqMan assay.

**Results:**

The pathogenic allele frequency for A allele was 0.03 (3%) and 0.07 (7%) for *FII* and *FV* genes, respectively. The GA/AA genotypes (c.*97G > A variant) were observed in only 33 (5.03%) individuals in the studied group. Additionally, the frequency of GA/AA genotypes was over 17.4% in the coagulation factor V. Co-incidence of heterozygous genotype GA of variants *FII* and *FV* genes was observed in only 4 subjects.

**Conclusion:**

The *FII* gene variant shown in our study is less frequent than in other European countries (about 6%). In contrast, the A allele of the *FV* gene occurs with a frequency similar to that of Northern, Central and South Central Europe (about 5%).

## Background

Thrombophilia can be defined as a predisposition to form clots inappropriately and may be inherited or acquired [1, 2] and this is the main factor promoting venous (rarely arterial) thromboembolism (VTE). VTE is a disorder of great importance to the health of general population; it has an incidence of 1 in 1000 persons per/ year [3]. The main clinical manifestation of VTE is a deep vein thrombosis (DVT; usually occurring in the legs) and pulmonary embolism (PE) which are responsible for substantial morbidity and mortality. According to data published in 2007, approximately 400,000 deaths per year in the European Union are a consequence of deep vein thrombosis [4]. In Poland, PE is responsible for about 30,000 deaths per year [5]. VTE is not only a risk factor for mortality (strokes, heart attacks), but it also causes morbidity post-thrombotic syndrome and chronic thromboembolic pulmonary hypertension (CTEPH) which reduce the quality of life for patients.

The acquired tendency to develop thrombophilia and VTE is connected with a lot of clinical or environmental-hypercoagulable states. The most commonly acquired thrombophilia factors are laparoscopic surgery, arthroscopic knee surgery, increasing age, obesity, smoking, immobility due to sitting and bed rest more than three days [6]. Other thrombophilia and VTE risk factors are malignancy, pregnancy or polycythemia rubra vera. Oral drug and transdermal contraceptives, hormone replacement therapy and chemotherapeutic drug – tamoxifen are a strong hypercoagulable states [6, 7].

Inherited thrombophilia is mainly associated with two pathogenic variants in V coagulation factor (*FV*) and the prothrombin genes. In the *FV* gene the presence of the Leiden mutation c.1601G > A (p.Arg534Gln;R506Q; rs6025; NM_000130.4) occurrs in 1–7% of people in Europe and is rare or absent in individuals of African, Japanese or native American origin. The prothrombin gene (coagulation factor II, *FII*) mutation is connected with thrombophilia c.*97G > A (previously designated as G20210A or 20210G > A; rs1799963; NM_000506.3) and is present in 0,7—4% of people in the general population [8, 9]. Both pathogenic variants occur most often in the heterozygous form (referred to as carrier state) together or separately. Patients with either the heterozygous *FV* Leiden or *FII* mutation are at a mild risk of thrombosis and 3.8 and 4.9 times, respectively more prone to a first blood clot. However, if the patient is the carrier of both heterozygous mutations, then the risk becomes higher and increases by up to 20 times. Homozygotous patients with *FII* and *FV* mutations are very rare in the general population [9]. A deficiency of antithrombin, protein C, protein S, plasminogen and dysfibrinogenemias are less in common inherited thrombophilias. All known genetic factors are present in about 25% of unrelated VTE cases and in about 63% of patients with a family history of VTE. These inherited thrombophilias have limited clinical significance in primary care but they may be important for patients with DVT or PE. Deep vein thrombosis is a major risk factor for the development of PE, which is a life-threatening condition. Thrombophilia is a multifactorial condition and only the mutual interactions between the environment and genes may lead to the development of clinical manifestations [4, 8, 9].

In respect to the above-mentioned, the aim of our study was to evaluate the frequency of two pathogenic variants in *FII* and *FV* genes as inherited thrombophilia within a group of Polish population in comparison with other described populations. In the current group we would like to assess the incidence of alleles and genotypes only as a population study without reference to the clinical features of patients. Performing a case–control study is the next step in our research among in Polish population.

## Methods

The study group was composed of 633 unrelated patients between the ages of 18 and 70, hospitalized at selected Departments of the Clinical Hospital of the Medical University of Bialystok: Department of Neurology and Department of Endocrinology, Diabetology and Internal Medicine. The blood samples were also collected from the patients of the Genetic Counseling Clinic. All patients’ samples were collected between 2015 and 2018. All participants were Caucasian. Table 1 presents demographical data of the studied individuals. The blood donors were asymptomatic in terms of thrombophilia occurrence as well as venous thromboembolism. The exclusion criteria included: blood relatives of the patient, individuals with known familial history of thrombophilia, oral estrogen contraceptives, hormone replacement therapy, pregnancy and the puerperium, major orthopedic surgery within the previous 12 months, trauma or fracture, smoking, obesity, cancer, immobilization, catheterization, and acute infection. The study have been performed in accordance with the Declaration of Helsinki and was approved by the local Bioethics Human Research Committee (Medical University of Bialystok) and informed written consent was obtained from all the participants.

**Table 1:** Characteristics of the study group

	Studied group (*n* = 633)
Gender	
Males	285 (45%)
Females	348 (55%)
Age (years)	
Mean ± SD	43,3 ± 11,2
BMI	
Mean ± SD	25,2 ± 2,6

The DNA for the molecular study was extracted from peripheral whole blood leukocytes by the Qiagen column kit. Genotyping of *FII* and *FV* variants was performed using the 7900HT Fast Real-Time PCR System (Applied Biosystems, USA). All single nucleotide polymorphisms (SNP) in the studied genes (rs1799963—c.*97G > A, rs6025 – c.1601G > A) were genotyped by TaqMan assay, SNP technology, from a ready-to-use human probes library (Applied Biosystems, Foster City, CA, USA) with the TaqMan Genotyping Master Mix (Applied Biosystems, Foster City, CA, USA) in a 20 µl reaction volume. The final concentration of genomic DNA for all samples in the experiment sample was 10 ng/µl. The reactions were carried out under the following conditions: 10 min at 95 °C for starting Hot-Start Taq polymerase activity, 40 cycles of 92 °C for 15 s and 60 °C for 1 min.

The statistical analysis was performed by the Stata 15 software. The expected frequencies were compared with the observed ones using the χ2 method (Hardy–Weinberg test).

## Results

The frequency of genotypes and alleles of coagulation factors *FII* (rs1799963) and *FV* (rs6025) in our studied population is shown in Table 2. Our study showed that the pathogenic variants in *FII* and *FV* genes were observed in 2.7% (A) and 7% (A) of the analyzed group of people, respectively. The pathogenic allele frequency in the studied group for A allele was 0.03 (3%) and 0.07 (7%) for *FII* and *FV* genes respectively (Table 2). The GA genotypes, separately for both variants, were observed in 5.1% (FII) and 14.3% (FV) of cases. The GA/AA genotypes of the c.*97G > A variant were observed in only 33 (5.03%) persons in the studied group. In the coagulation factor V variant, the genotypes GA/AA appeared with the percentage of 17.4%. Co-incidence of heterozygous genotype GA of variant *FII* and *FV* genes was observed in only 4 subjects (data not presented). The gender stratification of the group showed that the distribution of alleles and genotypes of all studied variants was similar for both females and males (Table 2). Analysis of the genotype frequencies distribution in the studied group was consistent with the Hardy–Weinberg equation (χ^2^, p > 0.05: c.*97G > A – 0.68, c.1601G > A – 1.60).

**Table 2:** Alleles and genotypes distribution in the study group

	Study group	Female	Male
FII	*N* = *633*	*N* = *348*	*N* = *285*
rs1799963			
GG	600 (94.8%)	330 (94.8%)	270 (94.7%)
GA	32 (5.1%)	17 (4.9%)	15 (5.3%)
AA	1 (0.2%)	1 (0.3%)	-
G	1232 (97.3%)	677 (97.3%)	555 (97.4%)
A^a^	34 (2.7%)	19 (2.7%)	15 (2.6%)
FV			
rs6025			
GG	546 (86.3%)	306 (87.9%)	240 (84.2%)
GA	86 (14.3%)	42 (12.7%)	44 (16.3%)
AA	1 (3.1%)	-	1 (6.7%)
G	1178 (93%)	654 (94%)	524 (91.9%)
A^b^	88 (7%)	42 (6%)	46 (8.1%)

Allele frequency: ^a^ 0.03 (A), ^b^ 0.07 (A)

## Discussion

The coagulation factor V variant is a single point mutation, described as a transition of G (guanine) to A (adenine) in 1601 nucleotide position in exon 10, causes changes in the protein chain; the substitution of arginine for glutamine (R506Q) [10, 11]. This pathogenic variant leads to the resistance of coagulation factor V to proteolytic inactivation by the activated protein C (APC), which is consequently related to a predisposition to thrombosis. Factor V Leiden is a poor risk factor of VTE when we compared it with the impact of natural anticoagulants deficiency, but it may be present in around 20% of patients with venous thrombosis [12]. The legitimacy of conducting a molecular test of Leiden mutation in patients without a personal or familial history of VTE is constantly discussed. Apart from the Leiden mutation study, the evaluation of the pathogenic variant in the prothrombin gene is equally important. Despite the fact that the G20210A mutation in the *FII* gene increases the risk of deep venous thrombosis to a lesser extent than the Leiden mutation, it occurs even in 6.2% of patients with venous thrombosis and about 2.3% of healthy patients. These two pathogenic variants in the *FV* and *FII* genes are together considered as a common cause of hereditary thrombophilia. The clinical utility of molecular testing of c.1601G > A and 20210G > A variants for hereditary thrombophilia may have predictive value for future thromboembolic events, both in patients with and without prior thromboembolic disease. It is corroborated by published cohort studies in which patients both with and without mutation are observed for the development of thromboembolism [13]. Genetic tests for thrombophilia and their clinical utility are included in medical practice in the context of the general risk of thromboembolism and possible risk as well as the benefits of treatment, mainly with anticoagulants. The decision regarding their implementation is related, among other things, to the general low incidence of thromboembolism in the general population, taking into account the interaction of environmental and genetic factors that determine the appearance of the disease. A relatively increased risk of thromboembolism occurs in people with familial hereditary thrombophilia [14]. The importance of genetic variants in connection with the increased risk of VTE in the *FII* and *FV* genes has been described in the introduction. Heterozygosity of c.1601G > A and 20210G > A variants is a strong risk factor for occurrence of the first clot and increases this risk by up to 20 times [15]. In our study population we showed that the GA genotypes, separately for both variants, were observed in 5.1% (*FII*) and 14.3% (*FV*) of cases. The simultaneous mutations of Leiden and in the prothrombin gene was found in only 4 subjects (0.64%).The pathogenic allele in *FV* (A) gene were observed in our analyzed group of people to the value of 7%. The previously published data in the Polish population by Alder et al. showed the incidence value of the variant c.1601G > A of the FV gene at 2.0% (0.02) [16]. The difference in the frequency of the examined allele between our result and the Alder study may have multiple causes. The selection of the test group, exclusion criteria, geographical conditions or ultimately the choice of test method may significantly influence difference in results. Notwithstanding the aggregation of their data with the current data (in our study *n* = 633 and in Alder study *n* = 1588- total *n* = 2221) indicates a frequency value for Poland of 4,5% [16]. The distribution of the A allele of the *FV* gene shown in our study places Poland, according to the study by Clark et al. in the group of European countries with a frequency above 3%, regardless of whether we add our result to the one previously published by Alder [16, 17]. These include Northern European countries such as Sweden (4. 5%), Norway (4. 2%), Denmark (3. 9%), U. K. (3. 7%) [17–20], and Central European countries like Germany (3. 8%), The Czech Republic (5. 1%), Italy (4%), Greece (3. 2%), Hungary (3. 8%) Austria (2. 8%) [17]. The Romanian value (8. 3%) was found to be significantly higher than in all European countries [21]. However, in Slavic countries a lower frequency of this allele was observed (< 3%)[a]. In countries such as Slovenia, Croatia, Bosnia and Herzegovina, Serbia and Montenegro, the distribution of c. 1601A ranged from 1. 5 to 2. 5% [22]. Also in the countries of the Eastern European bloc—Finland, Russia, Ukraine and Belarus the frequency of this variant is low (0. 6–2.4%) [22, 23]. Interestingly, in Western European countries such as France, Spain and Portugal, the frequency of the A allele is very similar to that of Slavic countries (2%, 0. 9%, 1. 2% respectively) [17]. The result of the frequency of the A allele *FV* gene differentiates our country in this respect from the group of Slavic countries to which Poland belongs. The number of individuals in groups and geographical conditions can influences the results obtained. In our study for variant 20210G > A in the *FII* gene, we observed a frequency of prevalence with the value 0.03. By way of comparison, in the Bosnian population, the A variant of the *FII* gene occurred at a frequency of 6%, 1% in the Saudi population and 5.4% in the Italian population [24–26]. No data on the distribution of this allele in other populations is available.

It should be emphasized that individuals who carry either or both variants of the C.1601G > A gene *FV* and 20210G > A of the *FII* gene may never develop the VTE symptoms due to the multifactorial nature of this disease. Only a combination of various risk factors along with genetic factors such as surgery, hospitalization with prolonged immobilization or estrogen therapy can lead to the provoking of a clinical manifestation of thrombophilia. Up to a certain age the carriers of pathogenic variants remain asymptomatic because the risk of VTE increases with age [27]. The question arises whether it is worthwhile conducting a study for inherited thrombophilia in healthy subjects ? Considering the fact that thrombosis is the cause of significant morbidity and mortality in the world and the main reason for VTE, the answer should be “yes”, but in a selected group of patients. The American College of Medical Genetics (ACMG) published a standards guide for laboratory testing for factor V Leiden and factor II c.*97G > A [28]. All recommendations are presented in Table 3.

**Table 3:** Recommendation of ACMG (American College of Medical Genetics and Genomics)

Main recommendation in patients:
1. before the age of 50 with first unprovoked VTE or with recurrent VTE
2. with VTE when the results of it may influence the treatment and clinical decisions
3. with at least two VTE in the family or VTE cases which occurred in relatives of the first generation at an early age
May be considered in several circumstances:
4. like smoking females under the age of 50 with a history of acute myocardial infarction
5. in siblings of individuals known to be homozygous for factor V Leiden or factor II c.*97G > A
6. in an asymptomatic pregnant woman or a woman considering pregnancy, with first degree relatives with unprovoked VTE or VTE caused by pregnancy or by use of contraception can be considered to undergo this test
7. in a pregnant woman’s family and a woman planning to conceive, the first degree relative who is the carrier of mutation Leiden and / or factor II c.* 97G > A and a history of VTE
8. in women who plan to start taking contraceptives or have hormone replacement therapy
The ACMG does not recommend routine testing for patients with a personal or family history of coronary artery disease or ischemic stroke

## Conclusion

This study showed the distribution of alleles and genotypes of two main pathogenic variants associated with inherited thrombophilia (*FII* and *FV*) within a group of unrelated Polish subjects. The frequency of A allele of the *FII* gene was 0.03 (3%). Compared to the described European populations, the frequency of this variant is relatively low but higher than in the African population (about 6% and 1% respectively). With regard to the A allele of the *FV* gene, it was present with a frequency of 7%. This result is similar to the distribution of this allele in the populations of North, Central and South–Central European countries with a value of about 5%. We are aware that the number of examined people in our sample is limited, however we do hope that our results may be helpful for future meta-analysis.

## Data Availability

All data are available from the corresponding author on request.

## Abbreviations

VTE: Thromboembolism
PE: Pulmonary embolism
CTEPH: Chronic thromboembolic pulmonary hypertension
APS: Antiphospholipid syndrome
FV: Coagulation factor V
FII: Coagulation factor II
SNP: Single nucleotide polymorphisms
G: Guanine
A: Adenine
APC: Activated protein C
ACMG: American College of Medical Genetics
